# Polycyclic Hydrocarbons Extracted from Tobacco: The Effect Upon Total Quantities found in Smoking

**DOI:** 10.1038/bjc.1956.78

**Published:** 1956-12

**Authors:** J. M. Campbell, A. J. Lindsey


### POLYCYCLIC HYDROCARBONS EXTRACTED FROM TOBACCO: THE EFFECT UPON TOTAL QUANTITIES FOUND IN SMOKING

J. M. CAMPBELL AND A. J. LINDSEY

*From the Department of Pathology, St. Bartholomew's Hospital, London, E.C.*1 *and the Department of Chemistry, Sir John Cass College, London, E.C.*3

Received for publication August 28, 1956

IN a recent investigation, the presence of polycyclic aromatic hydrocarbons in unsmoked Venda tobacco was demonstrated (Campbell and Cooper, 1955) and in consequence of these findings and in connection with other studies concerning the amounts of such compounds in pipe tobacco smoke (Gilbert and Lindsey, 1956) a series of experiments was carried out to determine the amounts in unburnt cigarettes, cigarette paper, pipe tobaccos and herbal smoking mixtures. These investigations are reported below.

### EXPERIMENTAL

All the materials examined were dried *in vacuo* over sulphuric acid and extracted to exhaustion with cyclohexane in a Soxhlet extractor. The solutions so formed were treated with acid and alkali and subjected to chromatographic separation and spectrophotometric determination by the method used for cigarette smoke (Cooper and Lindsey, 1955).

The smoking materials so examined were Virginia cigarettes of the type used in previous experiments, paper " cigarettes ", a light pipe tobacco, a dark brown highly aromatic pipe tobacco, a coarse herbal mixture for use in pipes and a fine herbal mixture for use in cigarettes. All these smoking mixtures were identical with those used in recent studies of pipe smoke (Gilbert and Lindsey, 1956). In Table I, column A, are shown the results of these determinations of extractable hydrocarbons.

The extracted materials were re-humidified by keeping them in a closed vessel over water until they had taken up water vapour to about 12 per cent, and they were then smoked in pipes (except for the cigarettes and paper " cigarettes ") as described in previous investigations. The polycyclic hydrocarbons were then determined in the smoke samples. These amounts also are shown in Table I, column B, and for comparison in column C, the amounts of the hydrocarbons previously determined in the smoke of un-extracted smoking materials are shown. All the quantities refer to 100 g. of material extracted or consumed in smoking.

Several interesting points are demonstrated in the table. Firstly it should be noted that pipe smoking of all mixtures, whether extracted or not, gives rise to much greater quantities of hydrocarbons than have been found in cigarette smoke. This point has been noted and discussed in connection with cigarette tobacco smoked in pipes (Gilbert and Lindsey, 1956). Secondly, the presence of poly-

TABLE I.—*Polycyclic Hydrocarbons in Micrograms from* 100 *grams*

| | Cigarettes | | | Paper | | | Pipe tobacco (light) | | |
|---|---|---|---|---|---|---|---|---|---|
| | A | B | C | A | B | C | A | B | C |
| Acenaphthylene | — | 0·3 | 5·0 | — | 3·3 | 8·3 | — | 2·0 | 29·1 |
| Phenanthrene | — | 13·0 | — | — | 10·9 | 4·3 | — | 80·0 | 297·0 |
| Anthracene | 0·1 | 2·2 | 10·9 | 0·1 | 4·0 | 16·9 | 0·3 | 58·0 | 110·0 |
| Pyrene | 10·3 | 3·3 | 12·5 | 1·4 | 9·2 | 17·0 | 19·5 | 40·0 | 75·5 |
| Fluoranthene | 10·6 | 2·3 | — | 2·1 | 8·6 | 16·9 | 12·6 | 15·0 | 14·7 |
| 3 : 4-Benzpyrene | 0·25 | 0·4 | 0·9 | 0·1 | 1·4 | 4·0 | 0·5 | 3·0 | 8·5 |

| | Pipe tobacco (dark) | | | Herbal pipe mixture | | | Herbal cigarette mixture | | |
|---|---|---|---|---|---|---|---|---|---|
| | A | B | C | A | B | C | A | B | C |
| Acenaphthylene | — | 8·9 | 596·0 | — | 8·2 | 136·0 | — | 6·5 | 87·0 |
| Phenanthrene | — | 840·0 | 665·0 | — | 180·0 | 104·2 | — | 400·0 | 240·0 |
| Anthracene | — | 88·5 | 198·0 | 2·0 | 55·0 | 162·0 | 0·5 | 81·0 | 7·8 |
| Pyrene | 78·0 | 72·0 | 122·0 | 12·0 | 57·0 | 55·3 | 20·5 | 45·0 | 62·9 |
| Fluoranthene | 77·0 | 57·0 | 167·0 | 15·8 | 21·0 | 104·5 | 21·5 | 58·0 | 37·1 |
| 3 : 4-Benzpyrene | 5·4 | 7·0 | 11·3 | 0·3 | 13·0 | 6·5 | 1·4 | 6·2 | 8·9 |

cyclic hydrocarbons in unsmoked material is demonstrated in all the materials studied. The amounts are usually smaller than those found upon smoking and it must be assumed, as has been demonstrated recently for 3 : 4-benzpyrene (Lyons, 1955), that all the hydrocarbons originally present are partially destroyed on smoking. However, no uniformity is discernible between the quantities removed by extraction and the amounts found upon smoking. This must be attributed to the varied conditions of combustion, especially in pipes, of materials of very variable composition and of different origin. The extraction process removes considerable amounts of substances other than hydrocarbons (e.g. carotene and lycopene) and these and other unknown materials are therefore not available for the smoking process in the extracted materials.

It has been suggested that atmospheric pollution may contribute towards the hydrocarbon content of unburnt tobaccos (Gilbert and Lindsey, 1956) and in order to make an assessment of the contribution from this source, a Virginia-type tobacco grown in Hertfordshire during the season 1954, and air-cured was also examined by the extraction process. The amounts of the various hydrocarbons found are shown in Table II, which also gives similar figures for fresh tobacco leaves flown from Rhodesia in a sealed cannister, and the Venda tobacco experiments described by Campbell and Cooper (1955). It will be noted that the English-grown tobacco upon extraction gives results closely similar to those given by the herbal mixtures which were mostly grown in England (Table I). It is also interesting to note that leaves freshly plucked show very small amounts of hydrocarbons ; presumably because there was no opportunity of contamination by smoke in Rhodesia.

TABLE II.—*Polycyclic Hydrocarbons in Micrograms extracted from* 100 *grams.*

| | English tobacco (air-cured) | Rhodesian tobacco (fresh leaves) | Venda tobacco |
|---|---|---|---|
| Anthracene | 0·2 | — | 1·0 |
| Pyrene | 10·1 | 1·1 | 3·0 |
| Fluoranthene | 15·2 | 0·5 | 11·0 |
| 3 : 4-Benzpyrene | 0·3 | — | 0·6 |

The examination of laurel leaves grown in smoke polluted areas has previously been used to measure atmospheric pollution (Coste, 1945). An independent test of the amounts of the polycyclic hydrocarbons taken up by leaf surfaces in England was therefore made by cutting leaves of the current years growth from the cherry laurel (*Prunus cerasifera*). These were collected on December 8, 1955 after a dry period of several weeks at three places ; one mile from Rickmansworth, Hertfordshire, a similar distance from Sevenoaks, Kent, and in Hyde Park, Central London. The smoke pollution was removed from the leaves by swabbing both sides with hydrocarbon-free cotton wool soaked in chloroform. The swabs were extracted with chloroform to exhaustion in a Soxhlet extractor, the dissolved material transferred to cyclohexane and the hydrocarbons determined by the method of Cooper (1954). The area of the leaves used was determined and in Table III the amounts of hydrocarbons found per unit area is shown.

TABLE III.—*Polycyclic Hydrocarbons Deposited on Cherry Laurel Leaves*

| | Micrograms per square metre | | |
|---|---|---|---|
| | Sevenoaks | Rickmansworth | Hyde Park |
| Anthracene | — | — | 1·3 |
| Pyrene | 2·9 | 2·4 | 16·3 |
| Fluoranthene | 3·8 | 3·3 | 12·2 |
| 3 : 4-Benzpyrene | 2·8 | 2·4 | 13·5 |
| 1 : 12-Benzperylene | — | — | 4·1 |

These quantities accord well with the proportions of smoke pollution found at the places named by snow analysis (Gilbert and Lindsey, 1955). No comparison can be made with the tobacco results since the leaf area of the sample grown in England could not be determined. It should be noted that more benzpyrene in proportion to the other hydrocarbons was found in the laurel experiments. Judging from the smoking materials grown in England (tobacco and herbal mixtures), an average of about 30 $\mu$g. total measurable hydrocarbons are extractable from 100 g. and an average of less than 1 $\mu$g. of this is 3 : 4-benzpyrene. If the results of Lyons (1955) may be applied to these very small amounts (a debatable matter) about 20 per cent of this passes into the mainstream smoke, and compared with the amounts found on smoking, this may be neglected.

The general conclusion from these experiments upon a most varied selection of smoking mixtures is that the smoking process makes the major contribution to the amounts of polycyclic hydrocarbons present in the mainstream smoke.

### SUMMARY

1. Polycyclic aromatic hydrocarbons have been extracted with cyclohexane from a number of smoking materials including cigarettes, various tobaccos, herbal smoking mixtures and cigarette paper.

2. The extracted materials, after re-humidifying, gave upon smoking further considerable amounts of hydrocarbons but these amounts were less than the amounts formed upon the smoking of the un-extracted materials. The smoking process produces the major amounts of the hydrocarbons.

3. It is concluded that atmospheric smoke pollution may in part account for the presence of these compounds in the smoking materials. Manufacturing processes may also have an effect, especially the blending of fire-cured tobacco in some mixtures. Tobacco leaves flown in sealed cannisters direct from a plantation in Rhodesia contained negligible amounts of polycyclic aromatic hydrocarbons.

The authors wish to acknowledge the support of the Medical Research Council and helpful criticism from Professor Sir Ernest Kennaway, F.R.S. They also wish to thank Mr. Lionel Henriques for collecting and sending fresh tobacco leaves from Rhodesia.

### REFERENCES

CAMPBELL, J. M. AND COOPER, R. L.—(1955) *Brit. J. Cancer*, **9**, 533.
COOPER, R. L.—(1954) *Analyst*, **79**, 573.
*Idem*, AND LINDSEY, A. J.—(1955) *Brit. J. Cancer*, **9**, 304.
COSTE, J. H.—(1945) ' Atmospheric Pollution in Leicester.' London. (H.M. Stationery Office), 151.
GILBERT, J. A. S. AND LINDSEY, A. J.—(1955) *Chem. & Ind.* (*Rev.*), 1439.—(1956) *Brit. J. Cancer*, **10**, 646.
LYONS, M. J.—(1955) *Rep. Brit. Emp. Cancer Campgn*, **33**, 278.


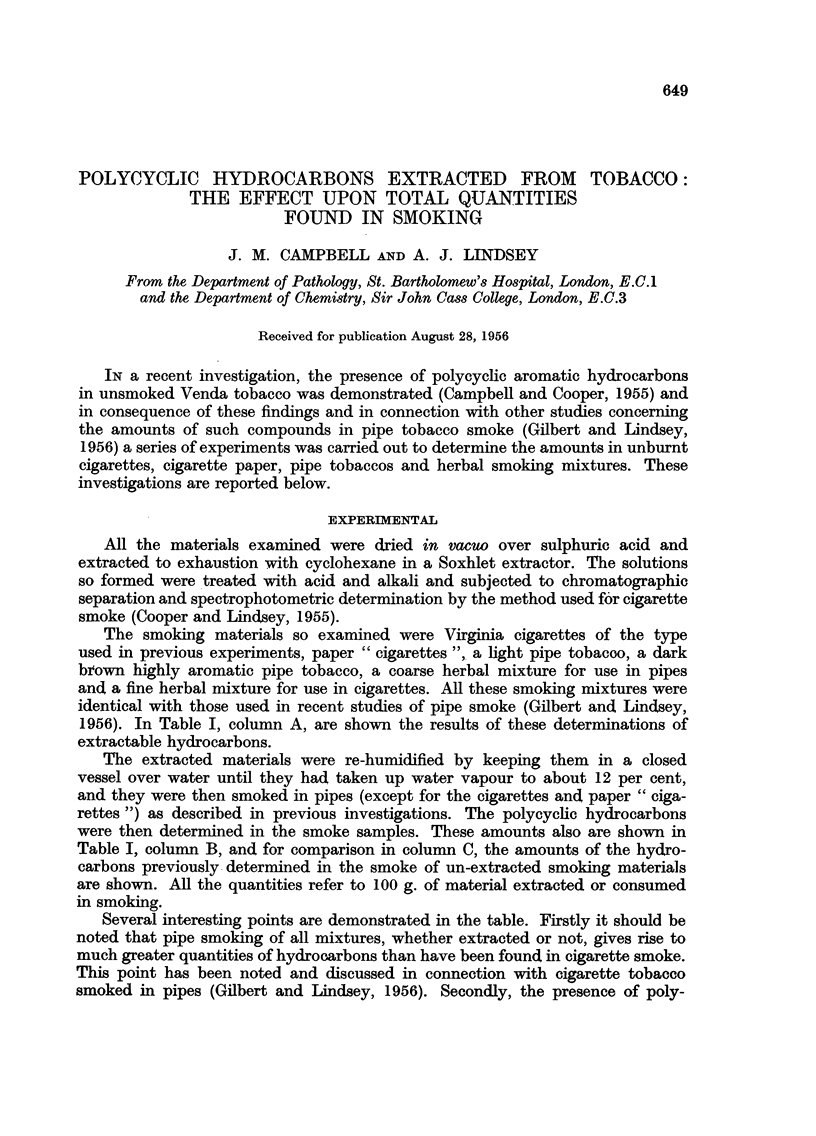

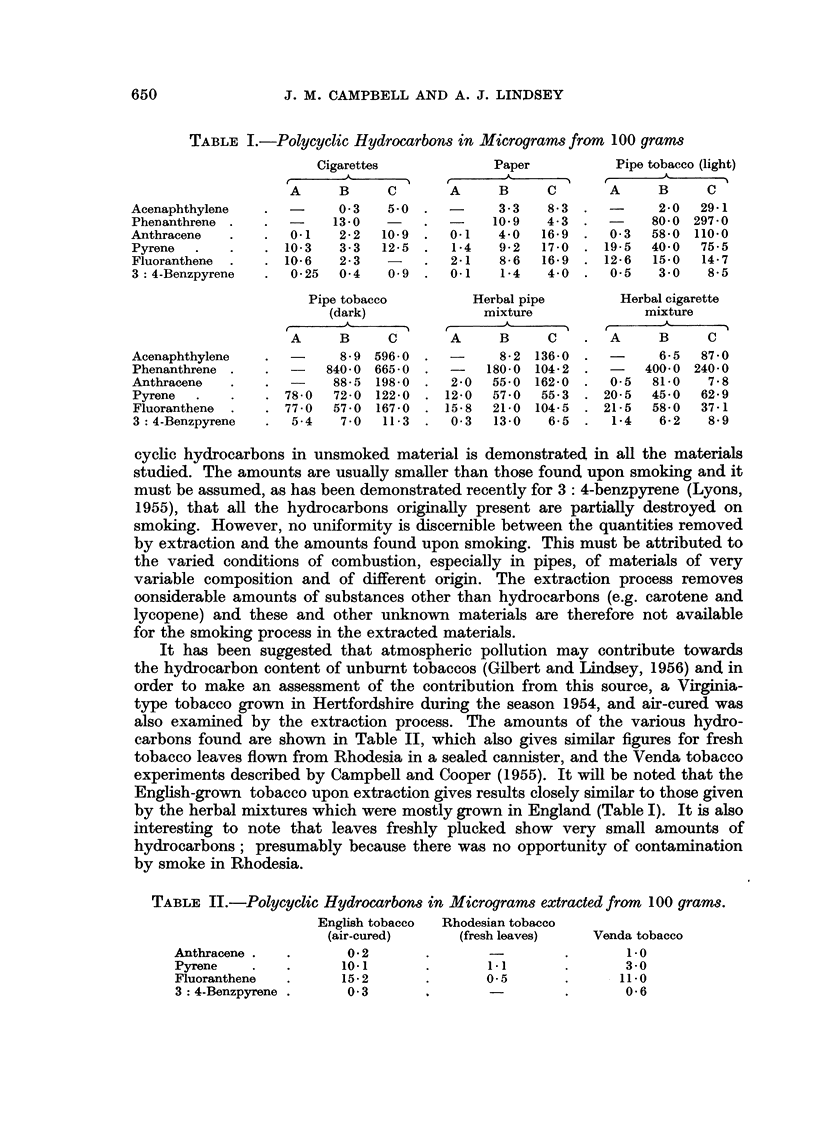

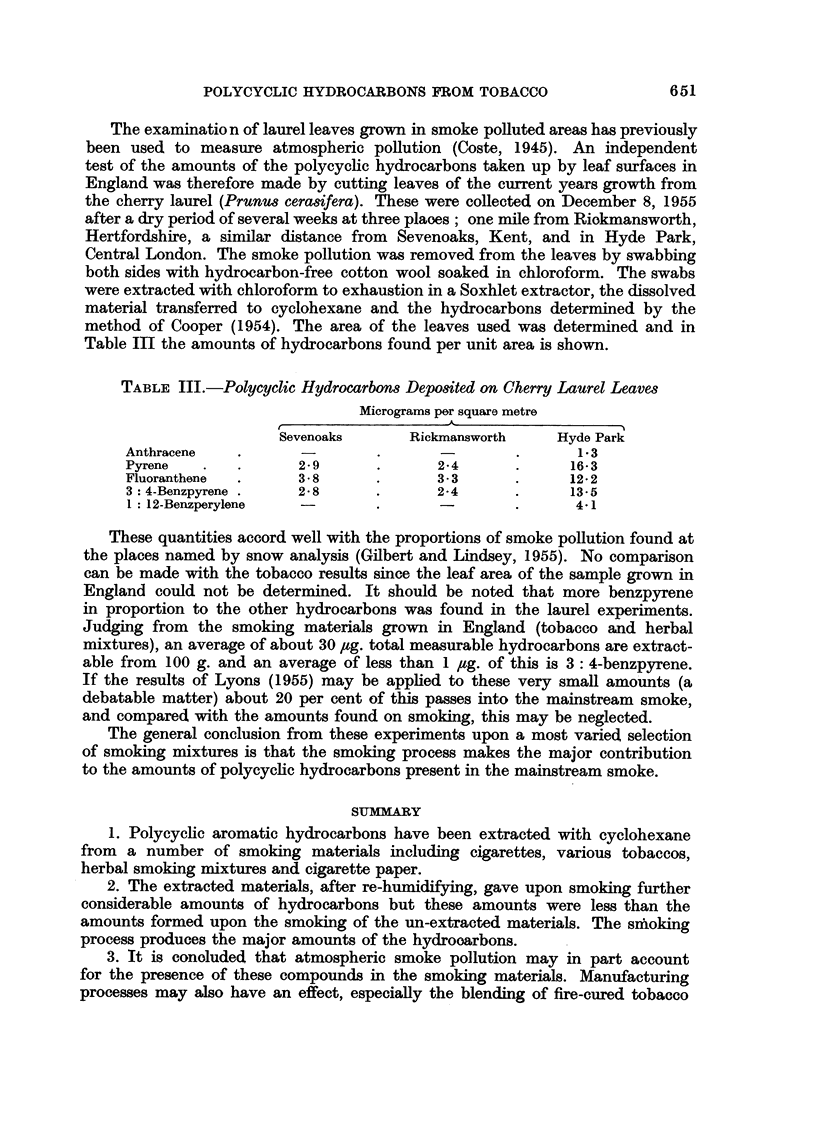

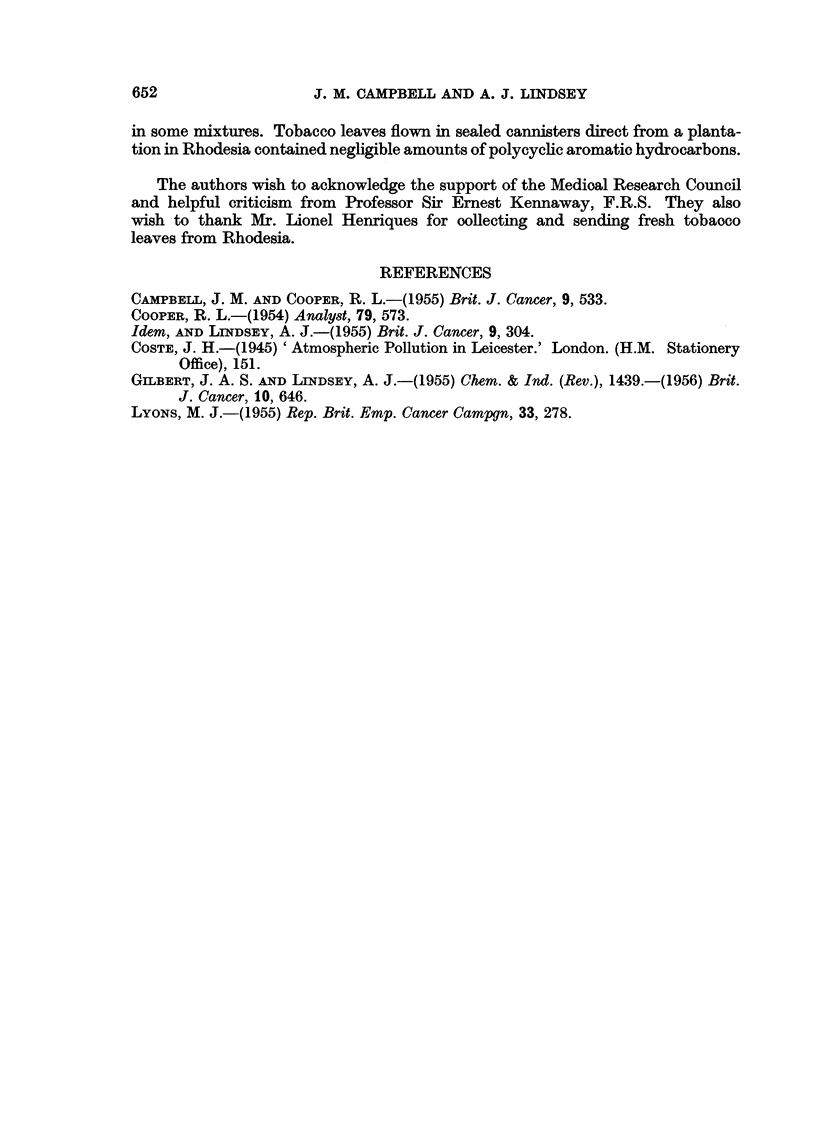


## References

[OCR_00295] GILBERT J. A., LINDSEY A. J. (1956). Polycyclic hydrocarbons in tobacco smoke: pipe smoking experiments.. Br J Cancer.

